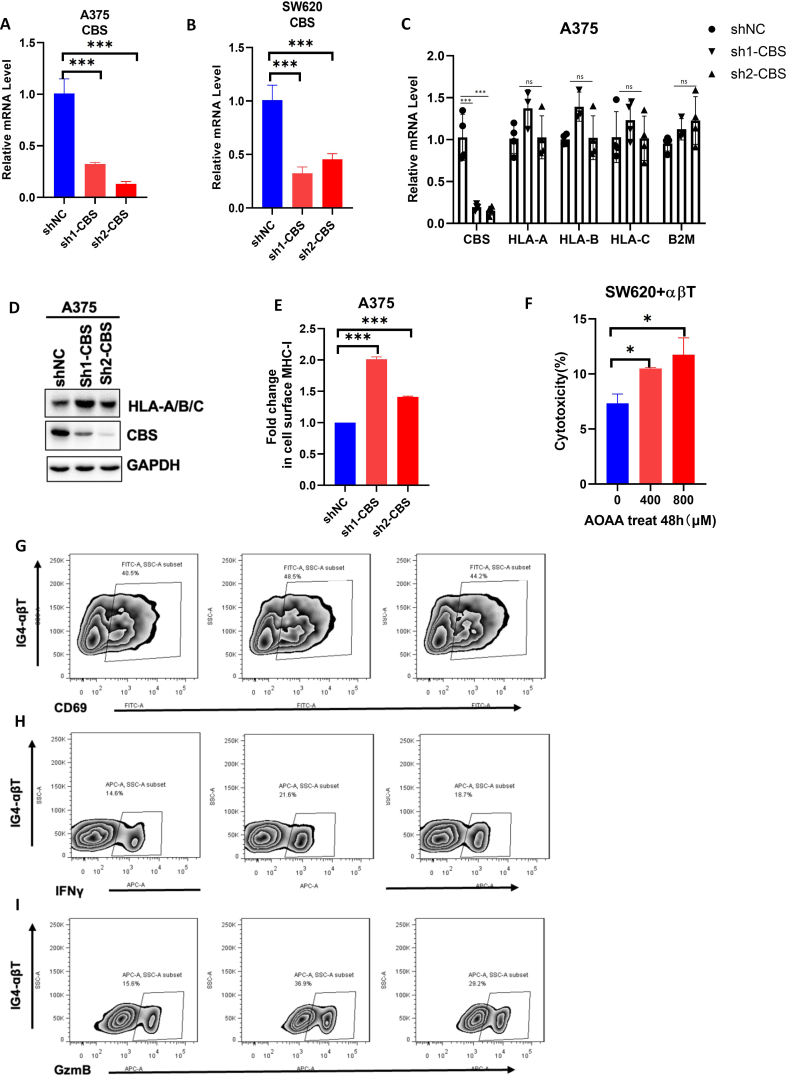# CBS promotes tumor immune evasion by reducing MHC-I stability

**DOI:** 10.1016/j.gendis.2025.101782

**Published:** 2025-07-25

**Authors:** Yanrong Yang, Yadong Guo, Shiyu Mao, Chengyuan Dong, Zhu Yu, Xudong Yao, Bing Shen

**Affiliations:** a Tongji University Cancer Center, Shanghai Tenth People's Hospital, Tongji University School of Medicine, Shanghai 200072, China; b Department of Urology, Shanghai Tenth People's Hospital, Tongji University School of Medicine, Shanghai 200072, China; c Urologic Cancer Institute, School of Medicine, Tongji University, Shanghai 200072, China; d Medical College, Anhui University of Science and Technology, Huainan, Anhui 232001, China; e Department of Laboratory Medicine, Shanghai Traditional Chinese Medicine-Integrated Hospital, Shanghai University of Traditional Chinese Medicine, Shanghai 201999, China

Cystathionine-β-synthase (CBS) is a key metabolic enzyme traditionally associated with homocysteine metabolism. Recent studies have uncovered its broader role in tumor biology,^1^ yet its impact on the tumor immune microenvironment remains largely unexplored. Tumor immunotherapy has rapidly advanced, with strategies such as immune checkpoint inhibitors, chimeric antigen receptor-T cell therapy, and tumor vaccines revolutionizing cancer treatment. Extensive analyses have summarized these approaches and their underlying mechanisms.^2^ However, metabolic regulators like CBS may also contribute to tumor immune escape, offering novel therapeutic targets. Here, we report for the first time that CBS promotes tumor immune evasion by destabilizing major histocompatibility complex class I (MHC-I) molecules, revealing a previously unrecognized link between tumor metabolism and immune surveillance.

Through The Cancer Genome Atlas (TCGA) analysis, we identified elevated CBS expression across multiple cancer types, including breast cancer, head and neck cancer, lung cancer, kidney papillary cell carcinoma, and prostate cancer (Fig. 1A). Protein-level validation confirmed significantly higher CBS expression in breast cancer and lung cancer (Fig. S1A–S1C), supported by immunohistochemistry (Fig. 1B; Fig. S1D). Receiver operating characteristic analysis demonstrated that CBS expression could serve as a pan-cancer diagnostic marker (Fig. S1E). Single-cell RNA-sequencing data visualized via uniform manifold approximation and projection (UMAP) effectively distinguished different cell types based on gene expression profiles (Fig. 1C). In cholangiocarcinoma (GSE138709), CBS was predominantly expressed in malignant cells (Fig. S1G). Compared with CBS-negative groups, CBS-positive groups displayed a higher proportion of malignant cells but lower CD8^+^ T-cell infiltration (Fig. 1D). Kruskal–Wallis analysis confirmed that CBS was primarily expressed in tumor cells (*p* < 0.05) (Fig. S1J). Spatial transcriptomics localized CBS expression to tumor cell regions (Fig. 1E; Fig. S1H and S1I), with the highest levels observed in malignant regions of ovarian cancer (Fig. 1F). Furthermore, CBS expression correlated spatially with tumor microenvironment components (Fig. 1G). Univariate Cox survival analysis linked CBS expression to increased overall survival in skin cutaneous melanoma (95% CI: 1.012–1.165; *p* < 0.05) and poor prognosis in adrenocortical carcinoma, kidney renal clear cell carcinoma, lower-grade glioma, uterine corpus endometrial carcinoma, and uveal melanoma (*p* < 0.05) (Fig. 1I; Fig. S1F). CBS expression was also inversely correlated with immune infiltration across multiple cancers (Fig. S1K). Single-sample gene set enrichment analysis (ssGSEA) revealed a significant negative association between CBS expression and antigen presentation pathways (Fig. 1H; Fig. S1L). Additionally, CBS-high skin cutaneous melanoma tissues exhibited significantly reduced CD8^+^ T-cell infiltration (Fig. S1M). In analyzing anti-programmed cell death ligand 1 (PD-L1) therapy response, we observed significantly lower CBS expression in responders within the Dizier cohort (Fig. 1J). Receiver operating characteristic analysis identified CBS as a strong predictor of melanoma immunotherapy response (the area under the curve: 0.857 (95% CI: 0.714–0.963)) (Fig. 1K). In the immunotherapy dataset, the CBS-high group exhibited worse progression-free survival (Fig. 1L) and overall survival (Fig. 1M). Given the negative correlation between CBS expression and antigen presentation, we investigated whether CBS regulated MHC-I and facilitated immune evasion. Using CBS knockdown models in A375 melanoma and SW620 colorectal cancer cells (Fig. S2A and S2B), we observed significant up-regulation of MHC-I proteins human leukocyte antigen-A (HLA-A), HLA-B, and HLA-C upon CBS knockdown (Fig. 1N; Fig. S2D), while their mRNA levels remained unchanged (Fig. 1O; Fig. S2C). Flow cytometry confirmed increased surface MHC-I expression following CBS knockdown (Fig. 1P; Fig. S2E). Aminooxyacetic acid hemihydrochloride treatment (CBS inhibitor) produced similar results (Fig. 1Q), suggesting that CBS negatively regulates MHC-I protein expression at the post-transcriptional level. To further explore how CBS affected changes in the total amount of MHC-I protein, we utilized the autophagy inhibitor bafilomycin A1 and the protein synthesis inhibitor cycloheximide to examine the effect of beta-1,3-N-acetylglucosaminyltransferase 2 (B3GNT2) on MHC-I stability. In CBS-knockdown cells, HLA-A/B/C levels declined at a slower rate in the absence of new protein synthesis (Fig. 1R). However, bafilomycin A1 treatment had minimal effect on HLA-A/B/C levels in CBS-knockdown cells (Fig. 1S). The results showed that down-regulation of CBS mainly enhanced MHC-I protein by slowing down MHC-I degradation.

**Figure 1:**
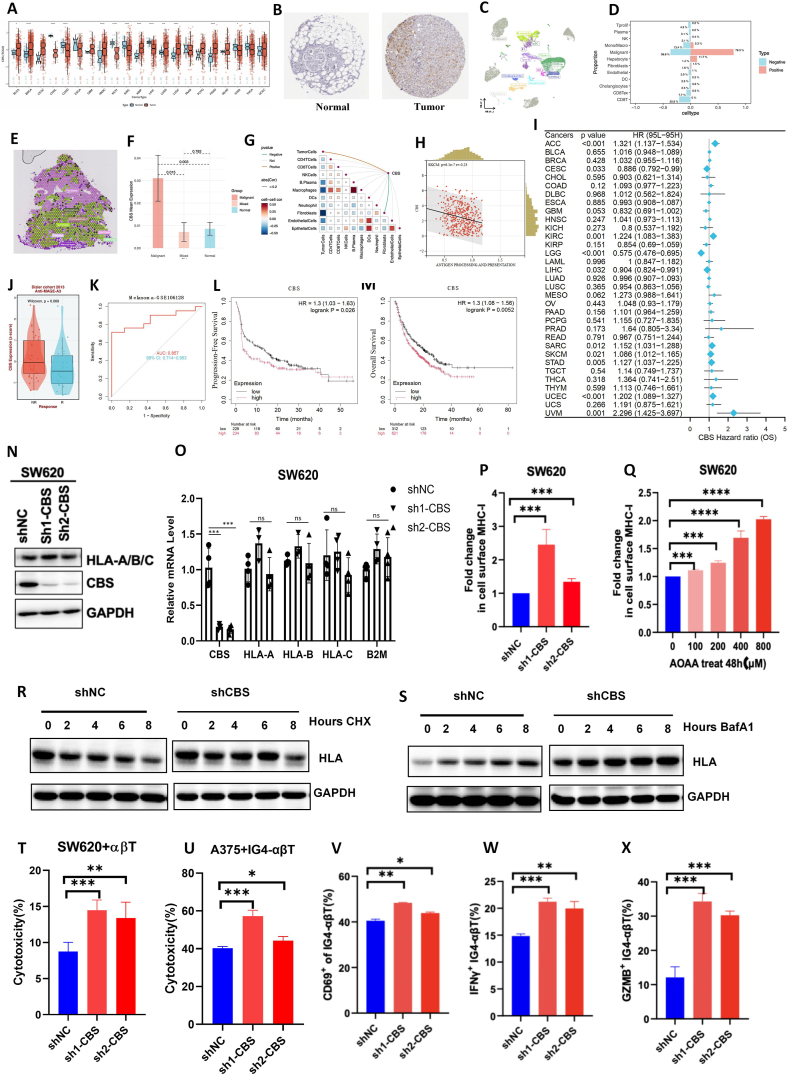
CBS facilitates tumor immune escape by destabilizing MHC-I. **(A)** CBS expression across various cancer types was analyzed using The Cancer Genome Atlas (TCGA) data. **(B)** Immunohistochemistry confirmed elevated CBS protein expression in breast cancer tissues. **(C)** Uniform manifold approximation and projection (UMAP) analysis of single-cell RNA-sequencing data (GSE138709) showed high CBS expression in malignant cells. **(D)** Comparison of malignant cell proportions and CD8^+^ T-cell infiltration between CBS-positive and CBS-negative groups. **(E, F)** Spatial transcriptomics illustrated that CBS expression was predominantly localized in tumor cell regions, with the highest levels observed in malignant areas of ovarian cancer. **(G)** Spatial correlation between CBS expression and components of the tumor microenvironment. **(H)** Single-sample gene set enrichment analysis (ssGSEA) demonstrated a negative correlation between CBS expression and antigen presentation pathways. **(I)** Univariate Cox analysis linked high CBS expression with increased overall survival (OS) risk in skin cutaneous melanoma (SKCM) and poor prognosis in adrenocortical carcinoma (ACC), kidney renal clear cell carcinoma (KIRC), lower-grade glioma (LGG), uterine corpus endometrial carcinoma (UCEC), and uveal melanoma (UVM). **(J, K)** CBS served as a predictor of anti-PD-L1 therapy response in the Dizier cohort. The value for the area under the receiver operating characteristic curve was 0.857, and there were higher response rates in the CBS-high group. **(L, M)** Kaplan–Meier survival analysis indicated worse outcomes for CBS-high groups in immune therapy datasets. **(N)** Western blotting analysis showed up-regulation of HLA-A/B/C protein levels following CBS knockdown in SW620 cells. **(O)** Quantitative PCR analysis revealed unchanged mRNA levels of HLA-A/B/C after CBS knockdown in SW620 cells. **(P)** Flow cytometry confirmed increased surface expression of MHC-I upon CBS knockdown. **(Q)** Aminooxyacetic acid hemihydrochloride (AOAA) treatment replicated the effects of CBS knockdown, enhancing MHC-I protein expression. **(R, S)** Cycloheximide (CHX) and bafilomycin A1 (BafA1) experiments confirmed that CBS regulated MHC-I stability rather than its synthesis or autophagic degradation. **(T)** Lactate dehydrogenase (LDH) release assay showed enhanced killing of SW620 cells by αβT cells upon CBS knockdown. **(U)** AOAA treatment similarly enhanced T-cell-mediated cytotoxicity against A375 cells. **(V**–**X)** Increased expression of CD69, IFNγ, and GzmB in T cells co-cultured with CBS-knockdown cells indicated heightened T-cell activation.

To assess CBS's impact on tumor immune evasion, we co-cultured CBS-knockdown SW620 cells with αβT cells and measured lactate dehydrogenase release. CBS knockdown significantly enhanced T-cell-mediated tumor cell killing (Fig. 1T), and similar effects were observed with aminooxyacetic acid hemihydrochloride treatment (Fig. S2F). To further verify the killing ability of antigen-specific T cells against CBS knockout cells, we modified the primary T cells to express a T cell receptor (IG4-αβT) that recognizes the specific antigen. The T cells specifically recognized the tumor antigen human cancer testicular antigen NY-ESO-1 (NY-ESO-1:157-165 epitope) and targeted to kill tumor cells that express HLA-A2 to present NY-ESO-1, such as human malignant melanoma A375. In A375 cells co-cultured with 1G4-αβT cells, CBS knockdown enhanced cytotoxicity, as evidenced by increased lactate dehydrogenase release (Fig. 1U). Additionally, flow cytometry of IG4-αβT functional factor interferon-gamma (IFNγ) or granzyme B (GZMB) showed that after T cells were co-cultured with A375 cells that knocked down CBS, IG4-αβT showed higher expression of IFNγ and GZMB (Fig. 1V–X).

Our findings establish CBS as a key regulator of tumor immune evasion, destabilizing MHC-I to impair antigen presentation and T-cell-mediated cytotoxicity. This extends CBS's known role beyond homocysteine metabolism, underscoring its significance in cancer biology. CBS has been implicated in tumor proliferation, metastasis, and therapy resistance, particularly in colorectal cancer and glioblastoma, through hydrogen sulfide (H_2_S)-mediated signaling.^3^ Additionally, it modulates redox balance and bioenergetics, enabling tumor survival under metabolic stress. Although its role in the tumor immune microenvironment remains underexplored, CBS may indirectly influence immune regulation by altering metabolic substrates and redox status, generating reactive sulfur species that disrupt immune cell function.

Our study fills this gap by demonstrating that CBS directly destabilizes MHC-I at the protein level, likely through post-transcriptional mechanisms. One potential pathway involves the ubiquitin-proteasome system, a primary route for MHC-I degradation. Given CBS's role in redox regulation, it may enhance oxidative stress, which in turn promotes MHC-I ubiquitination and accelerates proteasomal degradation.^4^ CBS-derived H_2_S or reactive sulfur species could modulate E3 ubiquitin ligase activity, further driving MHC-I degradation. Alternatively, CBS may promote autophagic turnover of MHC-I, as metabolic stress-induced autophagy has been shown to regulate MHC-I stability. CBS-induced metabolic reprogramming may activate key autophagy regulators, thereby promoting lysosomal degradation of MHC-I.^5^

Furthermore, CBS expression inversely correlates with immune infiltration across multiple cancers, suggesting its role in establishing an immunosuppressive microenvironment. Targeting CBS may enhance immunotherapy efficacy. Combining CBS inhibitors with immune checkpoint blockade, such as anti-programmed death-1 (PD-1)/PD-L1, could amplify T-cell activation and improve tumor clearance. Additionally, investigating CBS's interactions with immune regulators like PD-L1 and MHC-II, as well as its spatial distribution within tumors, may refine therapeutic strategies. Future studies should elucidate whether CBS directly interacts with MHC-I degradation machinery or modulates key ubiquitin ligases and autophagy regulators, offering deeper mechanistic insights into its role in immune escape.

Our study identifies CBS as a key regulator of tumor immune evasion, destabilizing MHC-I and impairing T-cell-mediated cytotoxicity. Targeting CBS offers a novel approach to enhance immunotherapy, particularly when combined with immune checkpoint inhibitors like anti-PD-1/PD-L1. By restoring MHC-I stability, CBS inhibitors may improve tumor response to T-cell-mediated killing, especially in CBS-high tumors with low immune infiltration. Future clinical trials should explore CBS inhibitors in combination with immune checkpoint inhibitors, focusing on patient stratification based on CBS expression. This strategy could offer new therapeutic opportunities to overcome immune escape and improve cancer treatment outcomes.

## CRediT authorship contribution statement

**Yanrong Yang:** Writing – original draft, Funding acquisition, Methodology, Investigation, Data curation, Validation. **Yadong Guo:** Writing – review & editing, Methodology, Validation, Formal analysis, Investigation. **Shiyu Mao:** Methodology, Investigation, Software. **Chengyuan Dong:** Methodology. **Zhu Yu:** Data curation. **Xudong Yao:** Visualization, Supervision, Validation, Resources, Writing – original draft, Project administration. **Bing Shen:** Validation, Software, Project administration, Conceptualization, Visualization, Writing – review & editing, Supervision, Resources.

## Data availability

The data are available from the corresponding author upon reasonable request.

## Funding

This work was supported in part by the China Postdoctoral Science Foundation (No. GZC20241242), the National Key Research and Development Program of China (2022YFB3804504); Cultivation grant for clinical and basicintegration research of Shanghai Tenth People's Hospital (SYYYRH-2025-034).

## Conflict of interests

The authors declared no conflict of interests.

## Figures and Tables

**figs1:**
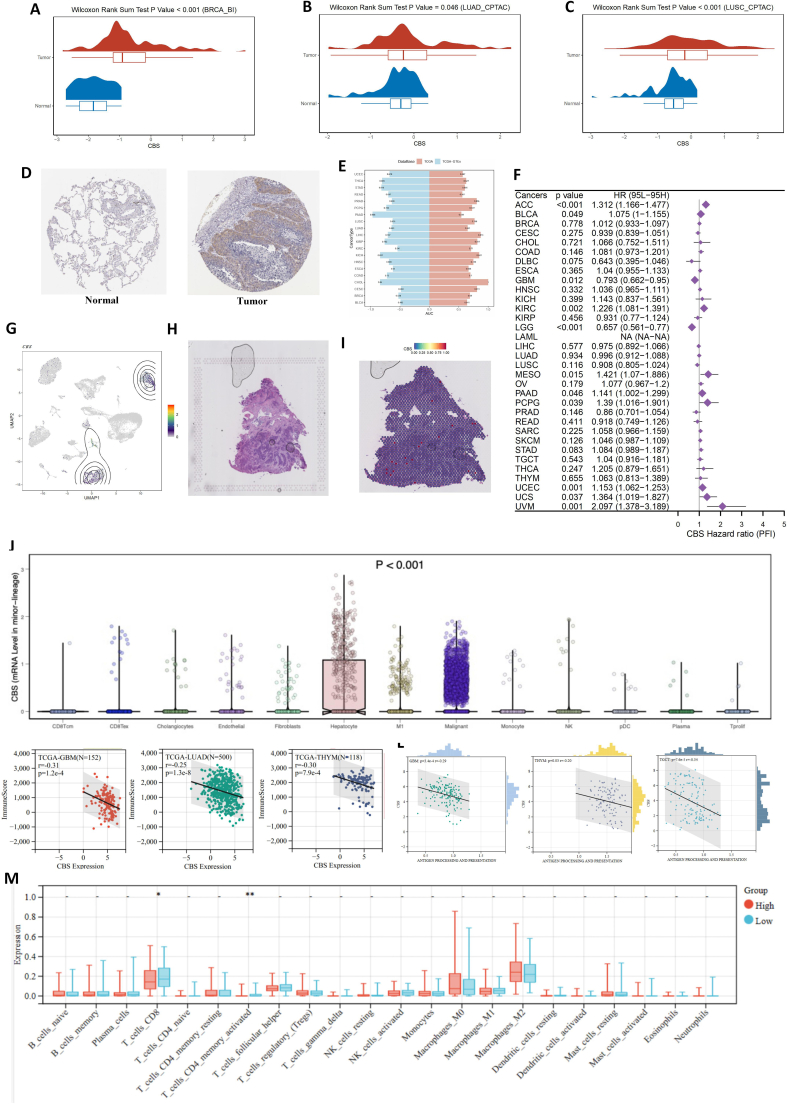


**figs2:**