# Supplementary material for: CBS promotes tumor immune evasion by reducing MHC-I stability
Source: Genes Dis. 2025 Jul 25;13(3):101782. doi: 10.1016/j.gendis.2025.101782 (PMC12854872; doi:10.1016/j.gendis.2025.101782)
Supplement: Multimedia component 1 [file mmc1.docx]

**Figure S1. CBS expression and its immune implications.**

(A-C) Western blot and IHC validating CBS overexpression at the protein level in BRCA and LCA.

(D) IHC results showing CBS expression in LCA tissues.

(E) ROC analysis identifying CBS as a pan-cancer diagnostic marker.

(F) Univariate Cox analysis associating CBS expression with higher progression-free interval (PFI) risk.

(G) UMAP analysis of single-cell RNA-seq data (CHOL, GSE138709).

(H-I) Spatial transcriptomics localizing CBS expression to tumor regions.

(J) Kruskal-Wallis analysis confirming predominant CBS expression in tumor cells.

(K) Negative correlation between CBS expression and immune cell infiltration across various cancers.

(M) Reduced immune cell infiltration observed in SKCM tissues with high CBS expression.

**Figure S2. CBS modulates MHC-I expression and T-cell-mediated immune responses.**

(A-B) Confirmation of CBS knockdown efficiency in A375 and SW620 cells.

(C) qPCR analysis showing no change in HLA-A/B/C mRNA levels following CBS knockdown in A375 cells.

(D) Western blot analysis demonstrating increased HLA-A/B/C protein levels upon CBS knockdown in A375 cells.

(E) Flow cytometry confirming enhanced surface MHC-I expression in CBS-knockdown A375 cells.

(F) AOAA treatment boosting T-cell-mediated cytotoxicity against SW620 cells.

(G-I) Elevated expression of CD69, IFNγ, and GzmB in T cells co-cultured with CBS-knockdown cells, reflecting stronger T-cell activation.

**Materials and Methods**

**Data Processing and Analysis**

We utilized publicly available datasets from The Cancer Genome Atlas (TCGA) project to analyze the expression characteristics of CBS across various cancers and its impact on patient prognosis. All data underwent standardization and preprocessing to ensure accuracy and consistency in the analysis.

1. **CBS Expression Analysis:**

CBS expression levels were evaluated using RNA-seq data, with differential expression analysis performed via the DESeq2 package in R. The expression differences between tumor tissues and adjacent normal tissues were compared.

1. **ROC Curve Analysis:**

The diagnostic performance of CBS in distinguishing tumor tissues from normal tissues was assessed using the pROC package. The area under the curve (AUC) and 95% confidence intervals (CI) were calculated, and smoothed curves were plotted.

1. **Survival Analysis:**

The effect of CBS expression on overall survival (OS) and Progression Free Interval (PFI) was evaluated using a Cox proportional hazards model. Univariate Cox regression was performed via the survival package in R, calculating the hazard ratio (HR) and 95% CI for each cancer type. HR > 1 indicated that high CBS expression increased mortality risk, while HR < 1 indicated reduced risk.

1. **Protein Expression Analysis:**

Differences in CBS protein expression between tumor and normal tissues in the BRCA, LUSC, and LUAD datasets were assessed using the Wilcoxon rank-sum test.

1. **Single-Sample Gene Set Enrichment Analysis (ssGSEA):**

The enrichment levels of specific gene sets in individual samples were evaluated using the GSVA package in R to perform ssGSEA.

1. **Immune Cell Composition Analysis:**

Immune cell subtype proportions were inferred using CIBERSORT, a deconvolution algorithm based on support vector regression (SVR), applied to mixed-sample gene expression data.

1. **Immunotherapy Response Analysis:**

Samples were divided into CBS high-expression and low-expression groups. Chi-square tests were used to compare immunotherapy response rates between the groups, while ROC curves were used to evaluate CBS expression’s diagnostic value in distinguishing responders from non-responders.

1. **Single-Cell RNA Sequencing Data Analysis:**

Single cell RNA-seq data from various cancers were analyzed by photothermal biological database (https://grswsci.top), and the data came from TISCH2 (http://tisch.comp-genomics.org/home/). Single-cell RNA-seq data from multiple cancers were analyzed using Seurat (v4.0.6) in R. Low-quality cells (fewer than 200 genes, >10% mitochondrial content, or potential doublets) were filtered. Data were normalized, scaled, and reduced via PCA and UMAP. Clustering was performed using Seurat’s Louvain algorithm (resolution = 0.5). Cell types and subpopulations were annotated based on marker genes from TISCH2, validated using SingleR. Differentially expressed genes were identified using FindMarkers (adjusted p < 0.05).

1. **Spatial Transcriptomics Analysis:**

Gene expression patterns were mapped onto tissue sections using spatial transcriptomics data, and spatially variable genes and their expression features were identified with SpatialDE software..

**Cell lines culture**

All cell lines were obtained from Cell Bank, Chinese Academy of Sciences (Shanghai, China) unless otherwise specified. HEK293T, and A375 cells were cultured in DMEM medium supplemented with 10% FBS. SW620 cells were cultured in RPMI 1640 medium supplemented with 10% FBS. PBMC cells were derived from peripheral venous blood from healthy volunteers, which were culture in RPMI 1640 medium supplemented with 10% FBS (with 200UI/ml IL2, 2nM Glutamine, 20µM β-mercaptoethanol, 100 U/ml penicillin and 100 μg/ml streptomycin) and the culture dishes were pre-coated with anti-human CD3 antibodies and anti-human CD28 antibodies for 37℃, 2h. All cells were maintained at 37℃ and in a 5% CO2 atmosphere. The cells were actively passaged for less than one month and were routinely tested for Mycoplasma contamination using the Myco-Blue® Mycoplasma Detector Kit (Vazyme, D101-01).

**Antibodies and Drugs**

APC anti-human HLA-A/B/C Antibody (311410/W6/32), Purified anti-human HLA-A/B/C Antibody (311402/W6/32), PE anti-mouse H-2 Antibody (125506/M1/42) were obtained from Biolegend. Anti-GAPDH antibody and anti- HLA class I ABC Polyclonal antibody were obtained from Proteintech. Anti-human CD3 antibody and anti-human CD28 antibody, Alexa Fluor® 647 anti-human/mouse Granzyme B Recombinant Antibody, APC anti-human IFN-γ, FITC anti-human CD69 Antibody were obtained from Biolegend. Cycloheximide (CHX, CAS No: 66-81-9) and Bafilomycin A1(BaFA1, CAS No: 88899-55-2) were obtained from MCE. AOA (Aminooxyacetic acid) hemihydrochloride (AOAA, CAS No: 2921-14-4) was obtained from Selleck.

**Cloning and transduction of shRNAs**

shRNAs were cloned into the pLKO.1 vector. Briefly, the target shRNA sequence is added to the following sequence, submitted to the company for single strand DNA synthesis and then annealed. Finally, the annealed PCR product was connected to the AgeI/EcoRI enzyme digested vector to obtain the final target plasmid. Forward oligo: 5’ CCGG—21bp sense—CTCGAG—21bp antisense—TTTTTG 3’. Reverse oligo: 5’ AATTCAAAAA—21bp sense—CTCGAG—21bp antisense 3’.

**Lentiviral Production and Transduction**

Lentiviral particles were generated by co-transfecting HEK293T cells with lentiviral transfer plasmids (shNC, sh1-CBS, or sh2-CBS) and the packaging plasmids pSPAX2 (Addgene #12260) and pMD2.G (Addgene #12259) at a ratio of 4:2:1 using Polyethylenimine Hydrochloride (PEI; Cat#24885-2, Polysciences). After 15 minutes of incubation at room temperature, the transfection mixture was added to HEK293T cells. Viral supernatants were harvested 48 hours post-transfection, filtered through a 0.45 μm filter, and used to infect target cells in the presence of 0.8 μg/mL polybrene. Forty-eight hours after infection, cells were selected with puromycin, and knockdown efficiency was confirmed by qPCR.

**Flow cytometry**

Tumor cells were transduced with the indicated shRNA and harvested. To avoid pipette errors, dilute the antibody by FACS Buffer (with 0.5% BSA) in a header in advance and mix well. Dilute antibody 50μL suspension cells were added to each sample. The cells were placed on ice and incubated for 30min away from light. During this period, to avoid cell formation and precipitation, tap the bottom of the EP tube every 10min. After the dyeing time, each tube was washed with 1mL FACS Buffer and centrifuged at 3000rpm for 5min. If only the protein on the plasma membrane was stained by flow cytometry, the washing FACS Buffer was discarded, and 200μL PBS was added to the cells to be suspended for machine detection. If the detection is not timely, it can be re-suspended with 2% paraformaldehyde, fixed for not more than one week. If further intracellular protein staining was needed, the washing FACS Buffer was discarded, and then 250μL fixing solution (BD) was added to each tube according to the fixing membrane breaking kit. The cells were suspended with the fixing solution and placed on ice for 30min. After completion, 500μL Washing Buffer (BD) was added to each tube, and the cells were mixed with a pipette and centrifuged at 4000 rpm for 5min. After the broken membrane was fixed, the intracellular protein staining process was homogenous membrane staining. Stained samples (barcoded or not) were analyzed or sorted on BD flow cytometers (Canto II, Fortessa, LSR II or ARIA II). Flow cytometry data was analyzed using FlowJo (Tree Star, Inc).

**T-cell isolation and NY-ESO-1 TCR-T cell construct**

Written informed consent was collected from participants after approval by the institutional review board. Peripheral blood mononuclear cells (PBMC) were isolated from heparinized whole blood of HDs by density gradient centrifugation over Ficoll/Hystopaque (Sigma-Aldrich). For αβT cell expansion: 500µL anti-human CD3 antibody (PBS dilution, 1:1000) was pre-coated in 24-well plates at 37°C for 2h. The coated antibodies were sucked out, washed with PBS, PBMC counted 2~3×106 antibodies/well into the well, and anti-human CD28 antibodies (direct addition: 1:400) were added into the well with 2 ml medium per well. After 48 hours of activation, the αβT cells were lentiviraly transduced with the NY-ESO-1 TCR to generate NY-ESO-1 TCR-T cells.

**T cell cytotoxicity assays**

Expanded T cells were thawed and maintained in culture media for 8–10 h before incubation with cancer cells. Cancer cells were suspended at 4 × 10^5^ cells/ml in culture assay medium and 50µl/well (20,000 cells) plated in 96 well-U-bottom plates before T cells were added at the appropriate effector to target cell (E:T) ratio. Then the plates were centrifuged 250g for 5mins and incubated at 37°C and 5% CO2 for 16-18h. Finally, the detection was carried out according to the instructions of the kit CytoTox 96® Non-Radioactive Cytotoxicity Assay (Promega, G1780).

**AOAA Inhibitor Treatment**

For inhibitor experiments, cells were seeded in 6-well plates and treated 24 hours later with aminooxyacetic acid (AOAA) at final concentrations of 0, 100, 200, 400, or 800 μM. After 48 hours of treatment, MHC-I expression was evaluated by flow cytometry or quantitative PCR (qPCR). For T cell cytotoxicity assays, cells were treated with AOAA at 0, 400, or 800 μM for 48 hours, followed by co-culture with αβ T cells.

**RNA extraction and quantitative PCR**

Total RNAs were extracted with TriZol (sigma) from various cells by RNA preparation kit as indicated. After their quantification using a Nanodrop 1000 spectrophotometer (Thermo Scientific), one microgram of total RNA was reverse-transcribed into complementary DNA using the ReverTra Ace qPCR RT Kit (Enzyme). Quantitative PCR was performed with SYBR Green Realtime PCR Master Mix (Enzyme) and quantified by the Step One Real-Time PCR System (Applied Biosystems). Primers for real-time PCR are shown as follows:

Actin-qPCR-F: ACACTGTGCCCATCTACGAG

Actin-qPCR-R: TCAACGTCACACTTCATGATG

CBS-qPCR-F: GAACCAGACGGAGCAGACAA,

CBS-qPCR-R: GTCGCTCAGGAACTTGGTCA,

HLA-A-qPCR-F: GCTCCCACTCCATGAGGTAT,

HLA-A-qPCR-R: AGTCTGTGACTGGGCCTTCA,

HLA-B-qPCR-F: ACTGAGCTTGTGGAGACCAGA,

HLA-B-qPCR-R: GCAGCCCCTCATGCTGT,

HLA-C-qPCR-F: GGACAAGAGCAGAGATACACG,

HLA-C-qPCR-R: CAAGGACAGCTAGGACAACC,

B2M-qPCR-F: CCACTGAAAAAGATGAGTATGCCT,

B2M-qPCR-R: CCAATCCAAATGCGGCATCTTCA.

**Western blot**

Western blot was conducted as follow: Cells were lysed in lysis buffer (50 mM Tris-HCl, pH 7.4, 150 mM NaCl, 1 mM EDTA, 1% Tritonx-100, 5% Glycerol and a cocktail of proteinase inhibitors). After lysis for 15min in 4°C, the soluble fraction of the cell lysates was isolated via centrifugation at 12,000 rpm in a microcentrifuge for 15 min at 4°C; After mixed with loading buffer, the proteins were processed at room temperature, resolved via SDS-PAGE gel electrophoreses, and analyzed via immunoblotting.

**Statistical Analysis**

All statistical analyses were conducted using R (4.1.0) and GraphPad Prism 8. The above bioinformatics analysis was completed by <https://grswsci.top.> Quantitative data were presented as mean ± standard deviation. Group comparisons were performed using two-tailed t-tests, and contingency table data were analyzed with Fisher’s exact test. Pearson correlation was used to assess relationships between variables. Survival analysis was conducted using Kaplan-Meier curves and the log-rank test. The significance level is defined as: *p < 0.05, **p < 0.01, *** p < 0.001; Ns means there is no statistical significance.
